# Weighted Correlation Gene Network Analysis Reveals New Potential Mechanisms and Biomarkers in Non-obstructive Azoospermia

**DOI:** 10.3389/fgene.2021.617133

**Published:** 2021-03-31

**Authors:** Meng Dong, Hao Li, Xue Zhang, Jichun Tan

**Affiliations:** ^1^Center of Reproductive Medicine, Shengjing Hospital of China Medical University, Shenyang, China; ^2^Key Laboratory of Reproductive Dysfunction Diseases and Fertility Remodeling of Liaoning Province, Shenyang, China; ^3^School of Life Sciences, China Medical University, Shenyang, China; ^4^Department of Laboratory Medicine, The First Affiliated Hospital of China Medical University, Shenyang, China

**Keywords:** non-obstructive azoospermia, immune infiltration, WGCNA (Weighted Gene Co-expression Network Analyses), hub gene, biomarkers

## Abstract

Non-obstructive azoospermia (NOA) denotes a severe form of male infertility, whose etiology is still poorly understood. This is mainly due to limited knowledge on the molecular mechanisms that lead to spermatogenesis failure. In this study, we acquired microarray data from GEO DataSets and identified differentially expressed genes using the limma package in R. We identified 1,261 differentially expressed genes between non-obstructive and obstructive azoospermia. Analysis of their possible biological functions and related signaling pathways using the cluster profiler package revealed an enrichment of genes involved in germ cell development, cilium organization, and oocyte meiosis. Immune infiltration analysis indicated that macrophages were the most significant immune component of NOA, cooperating with mast cells and natural killer cells. The weighted gene coexpression network analysis algorithm generated three related functional modules, which correlated closely with clinical parameters derived from histopathological subtypes of NOA. The resulting data enabled the construction of a protein–protein interaction network of these three modules, with CDK1, CDC20, CCNB1, CCNB2, and MAD2L1 identified as hub genes. This study provides the basis for further investigation of the molecular mechanism underlying NOA, as well as indications about potential biomarkers and therapeutic targets of NOA. Finally, using tissues containing different tissue types for differential expression analysis can reflect the expression differences in different tissues to a certain extent. But this difference in expression is only related and not causal. The specific causality needs to be verified later.

## Introduction

Azoospermia affects approximately 10 to 15% of males seeking medical care for couple infertility, and is considered the most serious manifestation of male infertility (Practice Committee of American Society for Reproductive Medicine in collaboration with Society for Male Reproduction and Urology, 2008; Wosnitzer et al., 2014). Azoospermia can be classified as obstructive azoospermia (OA), with normal spermatogenesis, or non-obstructive azoospermia (NOA), in which spermatogenesis is impaired (Jow et al., 1993). OA is caused by physical blockage of the male excurrent ductal system, which obstructs the transport of sperm and may occur in any area between the testicular net and the ejaculatory ducts. It accounts for one-third of azoospermia cases (Jow et al., 1993). OA may be congenital (e.g., congenital bilateral absence of the vas deferens, idiopathic epididymal obstruction) or acquired (e.g., vasectomy, infection, trauma, and iatrogenic injury) (Practice Committee of the American Society for Reproductive Medicine in collaboration with the Society for Male Reproduction and Urology, 2019). Unlike OA, NOA only rarely correlates with physical blockage; instead, it results from a range of abnormal testicular histopathological features (Jarow et al., 1989).

Although NOA accounts for almost 66% of patients with azoospermia, its etiology remains poorly understood, with only a limited number of studies exploring its genetic and molecular mechanisms (Hu et al., 2011; Krausz, 2011; Cerván-Martín et al., 2020). For sperm to successfully undergo cell meiosis, human spermatogenesis relies on the coordinated regulation of testis-specific genes (Zheng et al., 2019). Identifying potentially complex genetic and molecular causes of azoospermia is of vital importance for the diagnosis of NOA (Dorosh et al., 2013; Krausz and Riera-Escamilla, 2018; Cerván-Martín et al., 2020). Therefore, exploring different genetic abnormalities during spermatogenesis could point to potential diagnostic biomarkers and therapeutic targets of NOA.

Large-scale gene expression analysis based on microarray and high-throughput sequencing technology can detect changes at the transcriptional level for a large number of genes simultaneously, making it a great tool to investigate various diseases including azoospermia (Malcher et al., 2013; Zheng et al., 2019). In this study, we first screened through the microarrays deposited in the Gene Expression Omnibus (GEO) database to obtain genes differentially expressed between OA and NOA. Then, we performed functional enrichment analysis and immune infiltration analysis to identify possible biological functions and signaling pathways involved in OA or NOA onset. Finally, functional modules related to clinical traits were screened based on weighted gene co-expression network analysis (WGCNA), and candidate hub genes were selected. The present study aimed to expand our understanding of the molecular mechanism(s) of NOA.

## Materials and Methods

### Acquisition of Datasets

Data on mRNA expression profiling microarrays were acquired from the GEO DataSets^1^. The GSE9210 series included 47 NOA samples and 11 OA samples, the GSE145467 series contained 10 NOA samples and 10 matched OA samples, and the GSE45885 series consisted of 27 NOA samples with the corresponding clinical parameters. As this study involved only a bioinformatics analysis of the GEO DataSets, no ethical approval was required.

### Data Processing

The limma package in R was applied to screen for differentially expressed genes (DEGs), with | fold change| >2 and adjusted *P* < 0.05 considered as cutoff values (Ritchie et al., 2015). If fold change <−2. It means that this gene is low expressed in NOA. If fold change >2. It means that this gene is highly expressed in NOA. The genes that were aberrantly expressed in the GSE9210 and GSE145467 series were selected as the final DEGs.

### Functional Enrichment Analysis

Functional enrichment analysis of DEGs was carried out by analyzing Gene Ontology (GO) terms and the Kyoto Encyclopedia of Genes and Genomes (KEGG) pathways using the cluster profiler package in R (Yu et al., 2012). *P* < 0.05 was considered statistically significant.

### Immune Infiltration Analysis

The gene set variation analysis (GSVA) method, an explicit modeling phenotype approach in the enrichment scoring algorithm in R (Hanzelmann et al., 2013), was employed to analyze expression data. We collected 24 immune cell gene sets based on previously published articles (Subramanian et al., 2005; Thorsson et al., 2018).Specifically, the gene set identified by GSVA was used to analyze the distribution of 24 immune cells and evaluate immune infiltration in NOA (Bindea et al., 2013). Using correlation analysis, we analyzed the correlation among immune cells.

The calculation was supported by a *t*-test. Pearson test was used to evaluate correlations.

### Identifying Modules Associated With Clinical Traits

Weighted gene co-expression network analysis, an effective data-mining method in R suitable for weighted correlation network analysis, was used to produce a hierarchical clustering tree (dendrogram) of DEGs based on the hclust and dissTOM functions, as well as to generate module clustering (Langfelder and Horvath, 2008). WGCNA will select an appropriate threshold based on the dataset for dimensionality reduction analysis. It will evaluate the model based on each power value. When the model’s *R*^2^ reached 0.9 for the first time, this power value was chosen as the threshold for subsequent analysis. The relationship between these clustered modules and clinical parameters of NOA was determined by comparing the genes in the modules with the GSE45885 series. The specific analysis process is that after using WGCNA algorithm for dimensionality reduction clustering, we can get the score of each module in each sample. The optimized correlation analysis function “cor” in WGCNA was used. We analyzed the correlation between module scores and clinical characteristics. Correlation coefficient greater than 0.25 means there is an interaction relationship.

### Protein–Protein Interaction (PPI) Analysis

The Retrieval of Interacting Genes (STRING) database tool^2^ was used to build a protein–protein interaction (PPI) network (Szklarczyk et al., 2019). We put the genes in the candidate modules into the STRING database for subsequent protein interaction network construction. Interacting pairs with confidence scores >0.4 (medium confidence) were considered significant and retained and were then visualized by Cytoscape software (Bader and Hogue, 2003; Shannon et al., 2003). In the PPI networks, centisape, an app in Cytoscape, was used to calculate the connectivity degree. After obtaining the degree, we sort the genes by degree; the higher the degree, the higher the ranking. Then we selected the top five genes as hub genes.

## Results

### Identification of DEGs in OA and NOA

**FIGURE 1:**
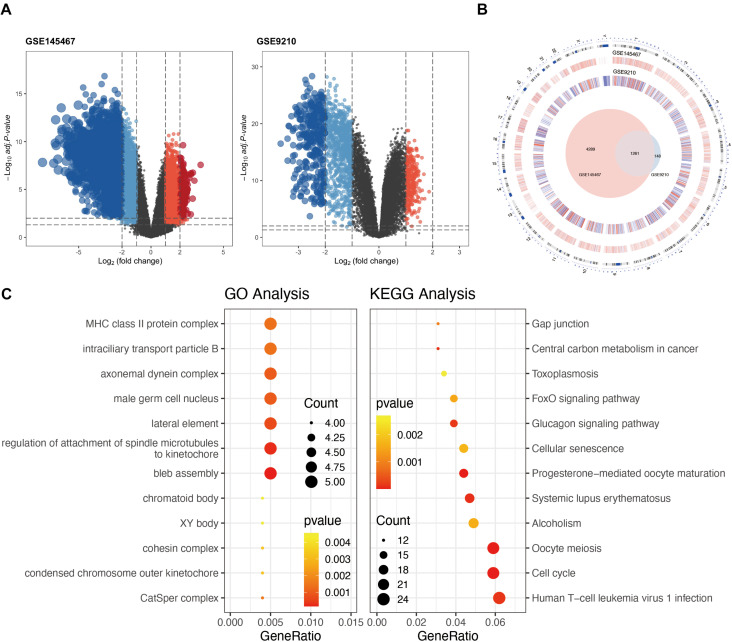
Analysis of differentially expressed genes (DEGs) in non-obstructive azoospermia (NOA). **(A)** Volcano plots of GSE145467 and GSE9210. Blue spots represent downregulated genes; red spots represent upregulated genes. Genes in black were not differentially expressed. **(B)** Circle plot of DEGs in both series. The outermost layer of the circle represents the chromosome location of the gene. The second and third levels indicate the positions of differential expression on chromosomes. Blue denotes downregulated genes; red denotes upregulated genes. The innermost Venn diagram represents the genes obtained after cross-analysis of the two series. **(C)** Enrichment of DEGs using Gene Ontology (GO) (left) or Kyoto Encyclopedia of Genes and Genomes (KEGG) (right) analysis. The larger the circle in the figure, the more genes it contains; lower *P* values are indicated with a stronger red color.

In the differential expression analysis, we considered NOA as case group. The high and low expressions mentioned in the analysis are all for NOA. Differential expression analysis identified 1,137 downregulated and 272 upregulated genes in GSE9210 (Supplementary Table 1), as well as 2,202 upregulated and 3,348 downregulated genes in GSE145467 (Figure 1A and Suplementary Table 2). Cross-analysis indicated the presence of 1,261 overlapping genes with stable differences (Figure 1B).

Functional enrichment on these 1,261 DEGs was performed to identify possible biological functions. GO analysis showed that NOA was related mainly to germ cell development, cilium organization, cilium assembly, nuclear division, spermatid development, and spermatid differentiation. When analyzing the related KEGG pathways, cell cycle, oocyte meiosis, progesterone-mediated oocyte maturation, human T-cell leukemia virus 1 infection, and FoxO signaling appeared to correlate with NOA (Table 1 and Figure 1C).

**TABLE 1:** Top 10 Enrichment analysis of the DEGs.

Ontology	ID	Description	Gene Ratio	*p* value	p. adjust	Count
BP	GO:0022412	cellular process involved in reproduction in multicellular organism	86/968	3.51E-36	1.68E-32	86
BP	GO:0007286	spermatid development	51/968	1.23E-30	2.96E-27	51
BP	GO:0007281	germ cell development	67/968	3.32E-30	5.30E-27	67
BP	GO:0048515	spermatid differentiation	51/968	1.54E-29	1.84E-26	51
BP	GO:0009566	fertilization	46/968	2.54E-20	2.43E-17	46
BP	GO:0044782	cilium organization	66/968	1.39E-19	1.11E-16	66
BP	GO:0051321	meiotic cell cycle	49/968	2.92E-19	2.00E-16	49
BP	GO:0060271	cilium assembly	64/968	4.63E-19	2.77E-16	64
BP	GO:0007338	single fertilization	39/968	6.59E-18	3.50E-15	39
BP	GO:0048285	organelle fission	67/968	4.37E-17	2.09E-14	67
CC	GO:0031514	motile cilium	56/1040	7.25E-30	4.04E-27	56
CC	GO:0097223	sperm part	57/1040	3.02E-28	8.41E-26	57
CC	GO:0001669	acrosomal vesicle	37/1040	8.00E-21	1.49E-18	37
CC	GO:0044441	ciliary part	70/1040	5.83E-17	8.12E-15	70
CC	GO:0000793	condensed chromosome	44/1040	2.01E-15	2.24E-13	44
CC	GO:0097014	ciliary plasm	28/1040	7.32E-13	6.79E-11	28
CC	GO:0005930	axoneme	27/1040	3.74E-12	2.98E-10	27
CC	GO:0005819	Spindle	52/1040	5.92E-12	4.12E-10	52
CC	GO:0097729	9 + 2 motile cilium	25/1040	9.02E-12	5.58E-10	25
CC	GO:0036126	sperm flagellum	24/1040	1.57E-11	8.77E-10	24
KEGG	hsa04110	Cell cycle	23/387	1.80E-08	4.89E-06	23
KEGG	hsa04114	Oocyte meiosis	23/387	3.37E-08	4.89E-06	23
KEGG	hsa04914	Progesterone-mediated oocyte maturation	17/387	4.10E-06	0.000396	17
KEGG	hsa05322	Systemic lupus erythematosus	18/387	8.42E-05	0.00537949	18
KEGG	hsa05230	Central carbon metabolism in cancer	12/387	9.50E-05	0.00537949	12
KEGG	hsa04913	Ovarian steroidogenesis	10/387	0.00012884	0.00537949	10
KEGG	hsa05166	Human T-cell leukemia virus 1 infection	24/387	0.00012985	0.00537949	24
KEGG	hsa04922	Glucagon signaling pathway	15/387	0.00015347	0.0055632	15
KEGG	hsa04540	Gap junction	12/387	0.00096965	0.02869978	12
KEGG	hsa04927	Cortisol synthesis and secretion	10/387	0.00098965	0.02869978	10

**TABLE 2:** Analysis of differential expression of immune infiltration between NOA and OA.

	Control	Case	*p* value
*N*	11	47	
aDC [mean (SD)]	0.06 (0.13)	−0.01 (0.14)	0.108
B cells [mean (SD)]	0.11 (0.06)	0.10 (0.05)	0.686
CD8 T cells [mean (SD)]	0.03 (0.05)	0.03 (0.03)	0.938
Cytotoxic cells [mean (SD)]	−0.06 (0.10)	0.15 (0.07)	<0.001
DC [mean (SD)]	−0.08 (0.05)	−0.00 (0.09)	0.009
Eosinophils [mean (SD)]	0.07 (0.06)	0.06 (0.08)	0.598
iDC [mean (SD)]	0.03 (0.09)	0.15 (0.04)	<0.001
Macrophages [mean (SD)]	0.13 (0.11)	0.26 (0.05)	<0.001
Mast cells [mean (SD)]	0.11 (0.06)	0.17 (0.06)	0.007
Neutrophils [mean (SD)]	0.05 (0.06)	0.17 (0.04)	<0.001
NKCD56bright cells [mean (SD)]	−0.15 (0.12)	−0.10 (0.09)	0.187
NK CD56dim cells [mean (SD)]	0.15 (0.07)	0.10 (0.07)	0.049
NK cells [mean (SD)]	−0.00 (0.05)	0.05 (0.03)	<0.001
T cells [mean (SD)]	0.08 (0.05)	0.13 (0.05)	0.002
T helper cells [mean (SD)]	0.22 (0.05)	0.11 (0.05)	<0.001
Tcm [mean (SD)]	0.14 (0.09)	0.07 (0.08)	0.014
Tem [mean (SD)]	0.02 (0.04)	−0.03 (0.05)	0.001
TFH [mean (SD)]	−0.12 (0.09)	−0.00 (0.06)	<0.001
Tgd [mean (SD)]	−0.30 (0.18)	−0.20 (0.41)	0.44
Th1 cells [mean (SD)]	0.10 (0.04)	0.15 (0.06)	0.016
Th17 cells [mean (SD)]	−0.03 (0.13)	0.06 (0.11)	0.021
Th2 cells [mean (SD)]	0.20 (0.05)	0.04 (0.09)	<0.001

### Infiltration of Immune Cells in NOA

**FIGURE 2:**
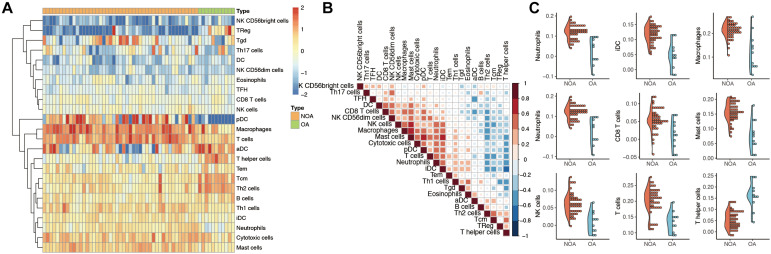
Evaluation of immune infiltration in non-obstructive azoospermia (NOA). **(A)** Heat map showing the expression of each immune cell in each sample. The horizontal represents each sample. The samples are sorted in the order of NOA and OA. The vertical is each immune cell. Through clustering, we visualize the clustering of immune cells with similar expression. **(B)** Correlation analysis among various immune cells. The red in the figure represents a positive correlation between the two immune components. Blue means there is a negative correlation between the two. The darker the color, the stronger the correlation. **(C)** Immune components associated with NOA. The curve generation distribution density in the figure. Scattered points represent the immune infiltration score of each sample.

The GSVA algorithm was used to analyze immune infiltration in NOA based on the distribution of 24 types of immune cells (Figure 2A). As shown in Figure 2B, there was a strong positive correlation between macrophages and mast cells (*R*^2^ = 0.707), as well as between macrophages and natural killer (NK) cells (*R*^2^ = 0.599). In contrast, a clear negative correlation was observed between interstitial dendritic cells (iDCs) and T helper cells (*R*^2^ = −0.627).

Moreover, we identified the immune components most clearly associated with NOA (Figure 2C). A comparison between NOA and OA case groups revealed that macrophages, neutrophils, NK cells, T helper cells, iDCs, and other immune cells were all significantly associated with NOA (Table 2).

### Module–Trait Correlations and Functional Enrichment Analysis

**FIGURE 3:**
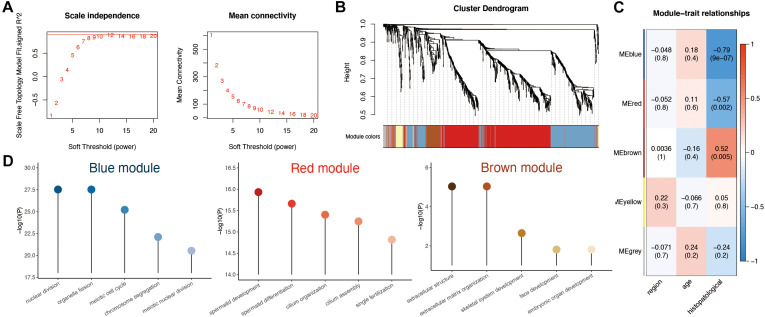
Weighted gene coexpression network analysis (WGCNA) analysis based on clinical characteristics in non-obstructive azoospermia (NOA). **(A)** Soft threshold selection in WGCNA; 10 as the final soft threshold for subsequent analysis. **(B)** Module cluster analysis of differentially expressed genes (DEGs). Cluster analysis of different genes. Group genes with similar expressions into one module. **(C)** Correlation analysis between modules and clinical characteristics. The number above represents the correlation coefficient, and the number below represents the *P* value. The red in the figure represents a positive correlation between the two immune components. Blue means there is a negative correlation between the two. The darker the color, the stronger the correlation. Correlation coefficient > 0.25 means there is an interaction relationship. **(D)** Enrichment analysis of clinical traits in the related modules.

To investigate the correlation between the above 1,261 DEGs and clinical traits of NOA, we conducted molecular clustering analysis on the GSE45885 series using the WGCNA algorithm. Three types of clinical traits were included in the GSE45885 series: age, localization, and histopathology subtype (postmeiotic arrest, meiotic arrest, premeiotic arrest, and Sertoli cell–only syndrome). According to an initial data evaluation, we selected a power value of 10 as an appropriate soft threshold for further analysis (Figure 3A). As a result, we obtained four related modules marked with different colors: blue (398 genes), brown (84 genes), red (625 genes), and yellow (48 genes) (Figure 3B).

To determine the clinical significance of the modules, we analyzed the correlation between the above four modules and clinical parameters. As shown in Figure 3C, none of the modules presented any significant relationship to age or localization. In contrast, the red and blue modules displayed a strong negative correlation with histopathological subtypes, whereas the brown module showed a positive association.

To better understand the possible biological function of the three modules associated with histopathological subtypes, we employed GO analysis. As shown in Figure 3D and Supplementary Table 3, genes in the red module were enriched in various ciliary parts, cilium organization, and cilium assembly; those in the blue module were related mainly to organelle fission, nuclear division, and chromosome segregation; finally, those in the brown module were enriched in extracellular structure organization, extracellular matrix, and skeletal system development.

### PPI Network Construction in the Modules

**FIGURE 4:**
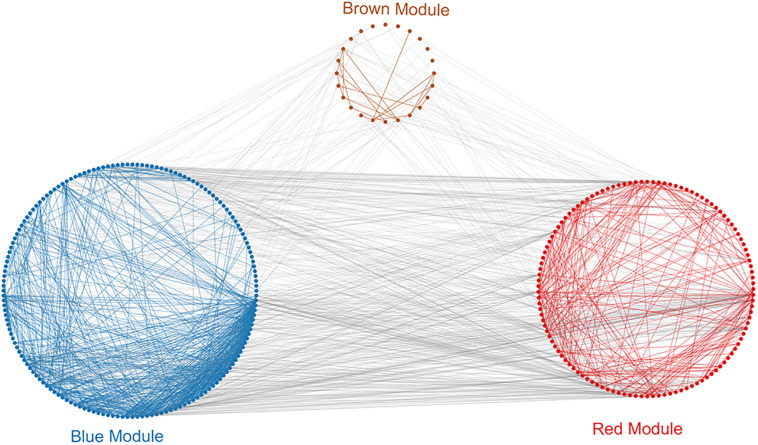
Protein–protein interaction (PPI) analysis of clinically relevant modules. First use the genes in the module to perform protein interaction analysis in the STRING database. Further build an interaction network between the overall related genes based on different module colors. Red spots represent the genes in the red module, blue spots denote those in the blue module, and brown spots denote those in the brown module.

To investigate in more detail the interplay between genes in the above three modules, we constructed a PPI network using the STRING database tool and 304 nodes (Figure 4). A screening for hub genes from the network indicated that the five genes with the highest degree of connectivity number were CDK1, CDC20, CCNB1, CCNB2, and MAD2L1 (Table 3). As shown in Table 3, all of the 5 hub genes were of low expression in NOA.

**TABLE 3:** Basic information of hub genes.

Name	Ensembl ID	Entrez ID	Description	Location	Degree	GSE9210 logFC	GSE145467 logFC
CDK1	ENSG00000117399	983	Cyclin Dependent Kinase 1	chr10:60,778,331-60,794,852	84	−1.48774115	−2.3920959
CDC20	ENSG00000117399	991	Cell Division Cycle 20	chr1:43,358,955-43,363,203	70	−1.26208441	−1.70986
CCNB1	ENSG00000134057	891	Cyclin B1	chr5:69,167,010-69,178,245	60	−1.04114957	−1.9196836
CCNB2	ENSG00000157456	9133	Cyclin B2	chr15:59,105,085-59,125,045	51	−2.16330599	−3.3849187
MAD2L1	ENSG00000164109	4085	Mitotic Arrest Deficient 2 Like 1	chr4:120,055,623-120,066,858	48	−1.26432724	−1.4974566

## Discussion

Azoospermia, the most intractable form of male infertility, is divided into obstructive (OA) and non-obstructive (NOA) azoospermia, which have distinct etiology and treatment (Berookhim and Schlegel, 2014; Bracke et al., 2018; Fainberg and Kashanian, 2019). Azoospermia not only can affect fertility, but also is correlated with higher incidence of other diseases such as tumors (Eisenberg et al., 2013). Because of the serious consequences of azoospermia, it is necessary to study the mechanism of this disease. Although it is essential to identify differences between the molecular mechanisms involved in both forms of azoospermia, very few such studies have been done so far. Here, we explored the molecular mechanism and potential diagnostic biomarkers in NOA.

First, we identified DEGs between NOA and OA, which yielded 1,261 overlapping genes with stable differences. We further performed a functional analysis of these DEGs. In order to understand the relationship between these related genes and clinical parameters. We used WGCNA for dimensionality reduction and correlation analysis. WGCNA can effectively detect coexpression modules and genes. Because of its extensive and powerful functions, this method has been widely used in various biological areas, to identify potential biomarkers or therapeutic targets (Fuller et al., 2007; Langfelder and Horvath, 2008; Zhao et al., 2010; Clarke et al., 2013; Huang et al., 2017; Liu et al., 2017).

These DEGs were found to be involved predominantly in germ cell development, nuclear division, and spermatid differentiation, raising the possibility that they may cause lack of sperm in the ejaculate. Moria et al. reported that aberrantly expressed genes such as RBM5 could lead to abnormal spermatid differentiation, germ cell sloughing, and apoptosis, ultimately resulting in the absence of sperm in the ejaculate (O’Bryan et al., 2013). Moreover, DEGs related to the function of ciliary parts, cilium organization or cilium assembly, might promote the occurrence of NOA. Although there is no evidence, we speculate that the reported increase in bacterial infection due to impaired ciliary function may contribute to azoospermia (Balbani et al., 2000). Therefore, the relationship between ciliary dysfunction and NOA should be scrutinized in future studies. Cell cycle and oocyte meiosis were the most significantly enriched pathways of DEGs in NOA. Indeed, DEGs can exert a non-negligible influence on each stage of meiotic progression, resulting in interrupted or incomplete spermatogenesis, or sperm maturation in this case (Dorosh et al., 2013; Long et al., 2017; van der Bijl et al., 2019; Yuan et al., 2019; Wyrwoll et al., 2020). In the pathway enrichment analysis, we have obtained multiple pathways related to NOA. The FoxO signaling pathway mainly regulates downstream genes by activating FoxO protein. This pathway can regulate processes such as apoptosis, cell cycle control, sugar metabolism, and oxidative stress resistance (Ponugoti et al., 2012). In the study of Ge et al., it was found that circRNA related to NOA is also involved in the regulation of FoxO signaling pathway. It shows that FoxO pathway is a key pathway of NOA (Ge et al., 2019). In the pathway analysis, a pathway similar to T-cell leukemia virus 1 was also enriched. There is no report on the study of NOA and T-cell leukemia virus 1. The results of the enrichment analysis can indicate that the genes related to NOA and T-cell leukemia virus 1 overlap, specifically whether NOA is related to T-cell leukemia virus 1. This requires follow-up research.

In the WGCNA analysis, genes in the blue module were significantly involved in nuclear division and chromosome segregation, whereas the genes in all modules were closely related to the occurrence of NOA. As discussed by He et al., in some infertile men, an altered crossover distribution was thought to disrupt the segregation of specific chromosomes (Ren et al., 2016). In the enrichment analysis of WGCNA-related modules, we found that the blue module is mainly related to meiosis, and the red module is mainly related to sperm formation. The brown module is mainly suitable for extracellular matrix organization. For the enrichment results of the red and blue modules, most of the enrichment results are related to NOA. However, there is no relevant research on the results of the brown module. This may be a direction for the next study of the NOA mechanism.

To explore the overall infiltration of immune cells in NOA, we used the GSE45885 series. Analysis of the distribution of 24 types of immune cells revealed that macrophages were one of the significant immune components in NOA, correlating positively with mast cells and NK cells. Using testicular biopsies of NOA patients, Goluza et al. reported substantial numbers of macrophages loaded with phagocytosed material, which is consistent with our bioinformatics analysis (Goluza et al., 2014). Macrophages have been visualized not only in the vicinity of seminiferous tubules, but also increasingly “intermingled” with Leydig cells. Our results suggest that mast cells and NK cells exerted a cooperative effect on macrophages, in line with previous reports on the interaction between macrophages and mast cells (Frungieri et al., 2002).

Finally, the PPI network built using the abovementioned three modules (blue, red, and brown), helped identify the following hub genes: CDK1, CDC20, CCNB1, CCNB2, and MAD2L1. We speculate that these genes may play an important role in histopathological subtypes of NOA. Cyclins (CCNB1, CCNB2) and cyclin-dependent kinases (CDK1) are key regulators of cell cycle, and consequently, they play an important role in meiosis and germ cell development (Chotiner et al., 2019). CDK1, in particular, was highly expressed, which could cause prolonged mitotic arrest during cell cycle in idiopathic NOA because of its extensive targeting for destruction through the APC/C^*CDC*20^ pathway (Li et al., 2017). CDK1 is required for meiotic progression to metaphase I in spermatogenesis, and while CDK1-deficient spermatocytes progress to the prometaphase stage, they then fail to reach metaphase I (Clement et al., 2015). Identified as a female infertility gene that initiates sister chromosome separation, CDC20 destroys cyclin B1 (Jin et al., 2010). CDC20 hypomorphic female mice, whether infertile or subfertile, produce aneuploid oocytes and embryos (Jin et al., 2010). CCNB1 and CCNB2 mRNA transcripts were found to be significantly reduced in patients with spermatogenesis disorders and might be considered as prognostic markers for azoospermic patients (Haraguchi et al., 2009). The MAD2L1 gene, which encodes the MAD2 protein, is a component of the mitotic spindle assembly checkpoint that prevents the onset of anaphase (Faisal and Kauppi, 2017). It is functional in both mitosis and meiosis (Lara-Gonzalez et al., 2012; Sun and Kim, 2012) and localizes to kinetochores in mouse spermatocytes (Kallio et al., 2000). Lower MAD2 levels have been found to reduce the apoptotic response to mis-segregating sex chromosomes and allow the formation of aneuploid sperm; hence, MAD2 levels are essential for the efficient elimination of aberrant spermatocytes (Faisal and Kauppi, 2017). Even though there have been only a few studies on the relationship between MAD2L1 and NOA, this gene and its protein product could be considered as potential biomarkers of NOA to focus on in future investigations.

In this article, we used the GEO dataset that contains very few patients with NOA/OA. The lack of sample size may cause some deviations in the analysis of our results. This is one limitation of our article. In addition, in the entire experimental design, NOA and OA may have some deviations in the results because of the difference in cellular complement.

In conclusion, we first performed differential expression analysis and enrichment analysis of between NOA and OA and found that NOA is mainly related to cell cycle, meiosis, and other processes. At the same time, the relationship between NOA and immune infiltration was evaluated. It is found that NOA is related to a variety of immune cells including macrophages. Finally, a key analysis of clinical characteristics was carried out through WGCNA to select key genes that affect NOA. Finally, it was found that CDK1, CDC20, CCNB1, CCNB2, and MAD2L1 are five important key genes. Our study provided useful hints as to which direction functional experiments should proceed.

## Data Availability Statement

The original contributions presented in the study are included in the article/Supplementary Material, further inquiries can be directed to the corresponding author.

## Ethics Statement

Ethical review and approval was not required for the study on human participants in accordance with the local legislation and institutional requirements. Written informed consent for participation was not required for this study in accordance with the national legislation and the institutional requirements.

## Author Contributions

MD: study design, bioinformatics analysis, drafted the manuscript, and contributed to the critical discussion. HL: bioinformatics analysis and manuscript revision. XZ: critical discussion. JT: study design, critical discussion, and manuscript revision. All authors have seen and approved the final version of this article.

## Conflict of Interest

The authors declare that the research was conducted in the absence of any commercial or financial relationships that could be construed as a potential conflict of interest.

## Footnotes

1 https://www.ncbi.nlm.nih.gov/gds/

2 string-db.org
